# Supplementary material for: Local and Systemic Endothelial Damage in Patients with CEAP C2 Chronic Venous Insufficiency: Role of Mesoglycan
Source: Int J Mol Sci. 2025 Apr 24;26(9):4046. doi: 10.3390/ijms26094046 (PMC12071570; doi:10.3390/ijms26094046)
Supplement: Supplementary file 1 [file ijms-26-04046-s001.zip › ijms-3555339-supplementary.pdf]

Supplementary materials

Supplementary materials, Table S1: Serum Levels of Parameters at baseline. Data are expressed as median and interquartile range in square brackets.

	Population (No. = 23)	
	Systemic Circulation T0	Varicose Vein T0
VCAM-1, ng/ml	709.9 [627.4-826.0]	763.5 [685.7-849.1]
ICAM-1, ng/ml	23.18 [18.43-28.55]	23.72 [21.64-29.65]
IL-6, pg/ml	2.57 [1.67-3.77]	3.89 [1.95-5.20]
IL-8, pg/ml	8.10 [4.91-17.96]	9.16 [6.86-14.76]
TGF-β, ng/ml	29.98 [21.98-44.32]	31.29 [20.55-46.15]
MMP-2, ng/ml	43.57 [37.23-79.19]	51.48 [46.94-88.39]
MMP-9, ng/ml	52.91 [36.87-94.26]	69.02 [39.89-96.74]
TIMP-2, ng/ml	129.56 [98.51-172.95]	127.29 [98.14-175.98]
SDC-1, ng/ml	177.2 [161.7-203.2]	187.4 [167.2-235.7]
SDC-4, ng/ml	51.74 [38.27-62.31]	56.75 [46.04-71.71]

Supplementary materials, Table S2: Serum Levels of Parameters in Varicose Vein at T1. Data are expressed as median and interquartile range in square brackets. Bold characters place emphasis on statistically significant values.

	Population (No. = 18)		
	Varicose Vein T0	Varicose Vein T1	*P*
**VCAM-1, ng/ml**	**763.5 [685.7-849.1]**	**660.6 [629.3-743.7]**	**0.001**
ICAM-1, ng/ml	23.72 [21.64-29.65]	25.95 [21.75-34.44]	0.89
**IL-6, pg/ml**	**3.89 [1.95-5.20]**	**2.53 [1.28-3.52]**	**0.006**
**IL-8, pg/ml**	**9.16 [6.86-14.76]**	**9.09 [6.86-13.90]**	**0.003**
TGF-β, ng/ml	31.29 [20.55-46.15]	25.33 [21.46-34.40]	0.631
**MMP-2, ng/ml**	**51.48 [46.94-88.39]**	**46.89 [40.92-66.78]**	**0.0002**
**MMP-9, ng/ml**	**69.02 [39.89-96.74]**	**36.22 [25.03-64.24]**	**0.003**
**TIMP-2, ng/ml**	**127.29 [98.14-175.98]**	**172.40 [118.97-211.29]**	**0.003**
**SDC-1, ng/ml**	**187.4 [167.2-235.7]**	**157.1 [140.5-179.4]**	**0.0005**

**SDC-4, ng/ml**	**56.75 [46.04-71.71]**	**42.22 [34.56-53.72]**	**0.0002**

Supplementary materials, Table S3: Serum Levels of Parameters in the Systemic circulation at T1. Data are expressed as median and interquartile range in square brackets. Bold characters place emphasis on statistically significant values.

	Population (No. = 18)		
	Systemic Circulation T0	Systemic Circulation T1	*P*
**VCAM-1, ng/ml**	**709.8 [627.4-826.0]**	**650.0 [587.5-727.0]**	**0.001**
ICAM-1, ng/ml	23.18 [18.43-28.55]	24.13 [21.74-32.56]	0.38
**IL-6, pg/ml**	**2.57 [1.67-3.77]**	**1.42 [0.97-3.18]**	**0.002**
**IL-8, pg/ml**	**8.10 [4.91-17.96]**	**8.45 [4.32-13.41]**	**0.001**
TGF-β, ng/ml	29.98 [21.98-44.32]	26.01 [23.52-32.98]	0.69
**MMP-2, ng/ml**	**43.57 [37.23-79.19]**	**42.84 [39.63-65.06]**	**0.0002**
**MMP-9, ng/ml**	**52.91 [36.87-94.26]**	**35.03 [19.88-75.61]**	**0.0003**
**TIMP-2, ng/ml**	**129.56 [98.51-172.95]**	**165.16 [122.59-197.94]**	**0.0002**
**SDC-1, ng/ml**	**177.2 [161.7-203.2]**	**148.5 [129.0-176.0]**	**0.0007**
SDC-4, ng/ml	51.74 [38.27-62.31]	37.07 [33.41-49.26]	0.065

Supplementary materials, Table S4: Comparison of Serum Level of Parameters at T1 between Systemic circulation and Varicose vein. Data are expressed as median and interquartile range in square brackets. Bold characters place emphasis on statistically significant values.

	Population (No. = 18)		
	Systemic Circulation T1	Varicose Vein T1	*P*
VCAM-1, ng/ml	650.0 [587.5-727.0]	660.6 [629.3-743.7]	0.138
ICAM-1, ng/ml	24.13 [21.74-32.56]	25.95 [21.75-34.44]	0.78
**IL-6, pg/ml**	**1.42 [0.97-3.18]**	**2.53 [1.28-3.52]**	**0.001**
**IL-8, pg/ml**	**8.45 [4.32-13.41]**	**9.09 [6.86-13.90]**	**0.006**
TGF-β, ng/ml	26.01 [23.52-32.98]	25.33 [21.46-34.40]	0.66
**MMP-2, ng/ml**	**42.84 [39.63-65.06]**	**46.89 [40.92-66.78]**	**0.03**
MMP-9, ng/ml	35.03 [19.88-75.61]	36.22 [25.03-64.24]	0.47
TIMP-2, ng/ml	165.16 [122.59-197.94]	172.40 [118.97-211.29]	0.27

**SDC-1, ng/ml**	**148.5 [129.0-176.0]**	**157.1 [140.5-179.4]**	**0.02**
**SDC-4, ng/ml**	**37.07 [33.41-49.26]**	**42.22 [34.56-53.72]**	**0.04**
